# Bayes Node Energy Polynomial Distribution to Improve Routing in Wireless Sensor Network

**DOI:** 10.1371/journal.pone.0138932

**Published:** 2015-10-01

**Authors:** Thirumoorthy Palanisamy, Karthikeyan N. Krishnasamy

**Affiliations:** 1 Department of Computer Science and Engineering, Nandha Engineering College,Erode, Tamilnadu; 2 Department of Information Technology, Sri Krishna College of Engineering and Technology, Coimbatore, Tamilnadu, India; Tianjin University of Technology, CHINA

## Abstract

Wireless Sensor Network monitor and control the physical world via large number of small, low-priced sensor nodes. Existing method on Wireless Sensor Network (WSN) presented sensed data communication through continuous data collection resulting in higher delay and energy consumption. To conquer the routing issue and reduce energy drain rate, Bayes Node Energy and Polynomial Distribution (BNEPD) technique is introduced with energy aware routing in the wireless sensor network. The Bayes Node Energy Distribution initially distributes the sensor nodes that detect an object of similar event (i.e., temperature, pressure, flow) into specific regions with the application of Bayes rule. The object detection of similar events is accomplished based on the bayes probabilities and is sent to the sink node resulting in minimizing the energy consumption. Next, the Polynomial Regression Function is applied to the target object of similar events considered for different sensors are combined. They are based on the minimum and maximum value of object events and are transferred to the sink node. Finally, the Poly Distribute algorithm effectively distributes the sensor nodes. The energy efficient routing path for each sensor nodes are created by data aggregation at the sink based on polynomial regression function which reduces the energy drain rate with minimum communication overhead. Experimental performance is evaluated using Dodgers Loop Sensor Data Set from UCI repository. Simulation results show that the proposed distribution algorithm significantly reduce the node energy drain rate and ensure fairness among different users reducing the communication overhead.

## Introduction

The major task of wireless sensor network (WSN) is intermittent monitoring of environment, where data or objects are sensed and then sent to the base or sink node for further processing. With the increasing demand in the network, the design of sensor nodes should be performed in such a manner with high-density operation with minimum transmission bandwidth. Therefore, the objective of minimizing network traffic has become an important issue.

Data Routing for In Network Aggregation (DRINA) for WSNs [1] resulted in reliable data aggregation with high aggregation rate. However, an effective balance between overhead and routing was not achieved. Cell Based Path Scheduling (CBPS) [2] used an algorithm based on zone pipeline scheduling with the objective of improving data collection. Sensed data communication was performed properly through continuous data collection, but at the cost of delay and energy consumption. Polynomial approximation algorithms were applied in [3] to address the issues related to delay with the aid of constrained and unconstrained version. Though numerical results proved computational complexity, weaker connectivity requirement remained unaddressed. In [4], with the objective of guaranteeing delivery, an efficient Beaconless Forwarder Planarization (BFP) scheme was introduced to improve the delivery rate.

The integrated Wireless Hospital Sensor Network [5] maximizes the lifetime of several medical data and operates cellular network resources. Each Body Area Network through formation of clusters consisting of Master Nodes creates minimum spanning trees (MSTs) with these clusters situated over the access points (APs) to limit the energy consumption of the medical Sensor Networks. Once the sensor nodes in wireless sensor networks are deployed, the nodes are unattended for a very long period of time. Due to this, occurrence of node failures, movement of nodes causes the network topology change over time. Coverage Inference Protocol (CIP) [6] was designed with the objective of minimizing the computation and communication overhead using time measurement. However, mechanisms for falsification of data were not addressed. A Dissemination approach based on hill climbing was designed in [7] to deal with increased changes in topology. Though average number of hops travelled by false reports were removed but at the cost of energy loss.

A new constructing approach for a weighted topology of wireless sensor networks (WSNs) is designed in [8] based on local-world theory for the Internet of Things (IOT). Based on local-world theory, an uneven clustering weighted evolving model of WSNs was designed. The definitions of edge weight and vertex strength take sensor energy, transmission distance, and flow are taken into consideration. However, the energy utilization remains unaddressed.

A scalable and energy efficient mechanism is designed in [9] to improve scalability and energy efficiency using context monitoring. Another topology controlled mechanism was designed in [10] for improving average throughput using Distributed Carrier Sense Update algorithm. But, optimality in data dissemination was not arrived. Dijkstra algorithm was introduced in [11] with single and multi-hop cluster chains with the motive of reducing the overhead.

In wireless sensor networks, each sensor nodes compete with each other in order to access the shared transmission medium. With increasing traffic in the network, proper routing protocols have to be designed due to the higher level of interference. Energy efficient method using energy balanced routing protocol is based on forward aware factor technique introduced in [12]. In FAF, the next hop was chosen on the basis of forward energy density and link weight. The forward aware technique was used as a communication protocol for balancing the energy consumption and therefore resulted in the increase in the network life time.

Maximum Weighted Matching (MWM) scheduling algorithm was designed in [13] to improve the throughput. However, the delay incurred was not considered. A biologically inspired approach was presented in [14] to reduce the collision occurring on heavy traffic loaded networks. A novel approach to map correlation of ID for RFID anti-collision was proposed in [15] to solve the problem of the lower efficiency. This method increased the association between tags so that tags can send their own ID under certain trigger conditions, by mapped correlation of ID, resulting in the efficiency of querying on multi-tree.

Among the various measurements the graph invariants have been utilized for the structure of entropy-based measures to describe the structure of complex networks in [16]. The Shannon’s entropy based graph measurement and vertex degrees effectively find their extreme values. However, the method did not analyze the discrimination power of this entropy. The molecular ID numbers were not suitable to generate the graphs because of their insufficient computational complexity. The uniqueness of Eigen value-based entropies was developed in [17] that provided efficient graph representation than the ID numbers. The unicyclic graphs with maximum energy were finally determined and the maximal energy among all bipartite bicyclic graphs using description of Hololumo index of graph was designed in [18].

This paper makes the following contributions: First, we present a Bayes Node Energy Distribution (BNED) model, which provides sink node an accurate subset of nodes by detecting target objects of similar events in an energy efficient manner reducing the energy consumption of sensor nodes. Second, we show that the major component of our BNEPD technique uses Polynomial Regression Function (PRF) to reduce node energy drain rate using polynomial coefficient. Therefore, the proposed BNEPD technique effectively integrates multiple functionalities like minimizing energy consumption, node energy drain rate with low time complexity. The performance of BNEPD technique compared with other possible data aggregations techniques is also investigated.

The rest of this paper is organized as follows: Section 2 introduces several routing mechanisms adopted in wireless sensor network. The core component of our technique called BNEPD technique and the design goals of it is included in Section 3. Section 4 includes the experimental setup with parametric definitions. Section 5 discusses in detail the results using table and graph form. Section 6 finally concludes with the concluding remarks.

## Related Works

Wireless Sensor Networks are equipped with different communication and computing capabilities and hence applicable to different operating scenarios. Hence routing protocol for relay node placement is one of the most important to be performed in WSNs involving diversified environments. In [19], approximation algorithms involving one-way and two-way was designed to reduce the number of relay nodes during transmission and thus ensuring throughput. However, mobility of relay nodes remained unaddressed.

Defending collaborative false data injection attacks in wireless sensor networks designed in [20] combine the keys of sensor nodes to their geographical locations, and validated the legitimacy of data by checking the locations of the sensors supporting the information. An optimal energy aware forwarding rule was introduced in [21] to control the motion using high mobile actors. But network lifetime maximization was not ensured. To maximize the lifetime of network, a packet forwarding scheme based on any cast mechanism was constructed in [22] using sleep wake scheduling principle. But, energy consumption involved during sleep wake remained unsolved. Energy aware clustering algorithms were introduced in [23] to not only increase the lifetime of the network but also to minimize the energy consumption.

One of the most important functionality considered in wireless sensor network is the link scheduling. This is because of the shared and wireless nature of the network that changes drastically over time. Wireless scheduling algorithms [24] used structural property to maintain queue length ensuring throughput. However, issues related with varying channels remained unsolved.

Active node and dynamic time slot allocation was introduced in [25] with the motive of reducing the energy usage on varying channels with the aid of Bit Map Assisted protocols. Another energy-aware routing scheme [26] used possible shortest hop paths in order to minimize the energy consumption. However, transmission power was compromised. The RNA shape software was used to calculate the minimum free energy structures of all sequences presents in the designed datasets and their equivalent shape described in [27]. Based on the above said methods, in this work, an efficient Bayes Node Energy Polynomial Distribution technique for efficient routing is designed in the forthcoming sections.

## Overview of Bayes Node Energy Polynomial Distribution (BNEPD) Technique

Wireless sensor network has been introduced for several reasons including securing the link to physical humanity, excellent robustness and capability to work; hence is employed for wide range of applications. In various WSN applications, motion strength has a common view of monitoring the field. Most of the existing researches on movement monitoring focused on dealing with single object, where object numeration was not required. Sensor nodes are used for sensing the node emitted by an object such as, sound, vibration, etc., to detect the appearance of an object, and then helps to monitor and track it.

**Fig 1:**
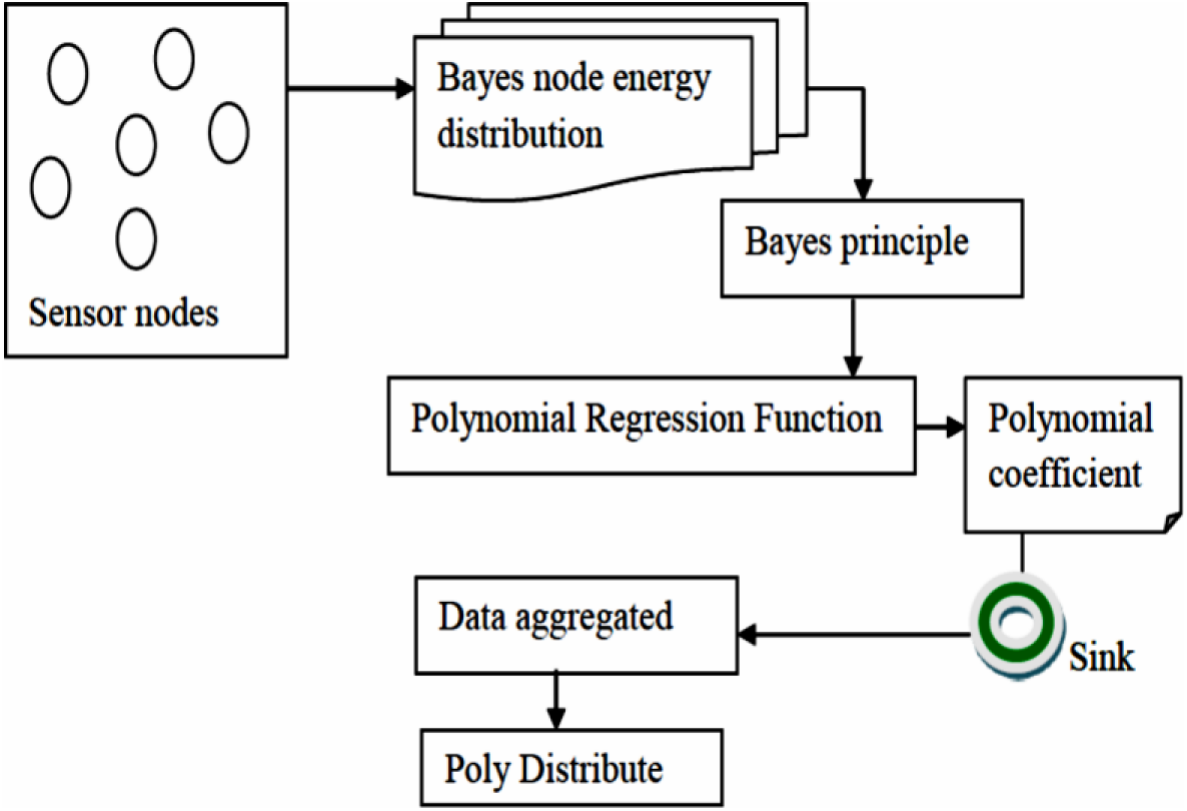
Bayes Node Energy Polynomial Distribution framework for WSN.

The proposed technique is employed to distribute the sensor nodes and identifies energy efficient routing path of similar event using Bayes Node Energy Distribution and Polynomial Regression Function in Wireless Sensor Network. The distribution of sensor nodes is performed through Bayes distribution that detects an object of similar event. The energy efficient routing path for each sensor nodes are generated by data aggregation at the sink based on polynomial regression function. We use a novel distribution algorithm to adaptively represent nodes stop sensing and transmitting data to sink for a specific time period. The framework of the Bayes Node Energy Polynomial Distribution technique is shown in Fig 1.

The framework of BNEPD technique is divided into construction of Bayes Node Energy Distribution, applying Polynomial Regression Function for data aggregation and application of Poly Distribute algorithm. The framework starts with the construction of Bayes Node Energy Distribution that applies Bayes rule through which the sensor nodes senses the target object of similar events (i.e., temperature, pressure) within its frequency to reduce the consumption of energy.

Next, the Polynomial Regression Function is applied to the target object of similar events for multiple sensors and are aggregated on the basis of the minimum and maximum object of events, sent to the sink node. Finally, the Poly Distribute algorithm distribute the sensor nodes in an efficient manner that splits the nodes to be in sleep state and nodes for object detection of similar events according to the number of nodes in the network and network size. The elaborate description of design of BNEPD technique is explained in the forthcoming sections.

### Bayes Node Energy Distribution (Reduces Energy Consumption)

In this section, a decentralized approach using Bayes principles is presented in order to increase the performance of the network. Instead of compressing data collected by the sensor nodes in Wireless Sensor Network, the BNEPD technique selects subset of the nodes (i.e., sense) in the network that detects an object of similar event and transmits the data to the sink node.

Whenever an additional sensor node has to be included in the network using conventional types of network, though results in increasing only a small portion of accuracy in the sensing file, due to highly correlated nature of the sensed data, but compromises the energy consumption level.

**Fig 2:**
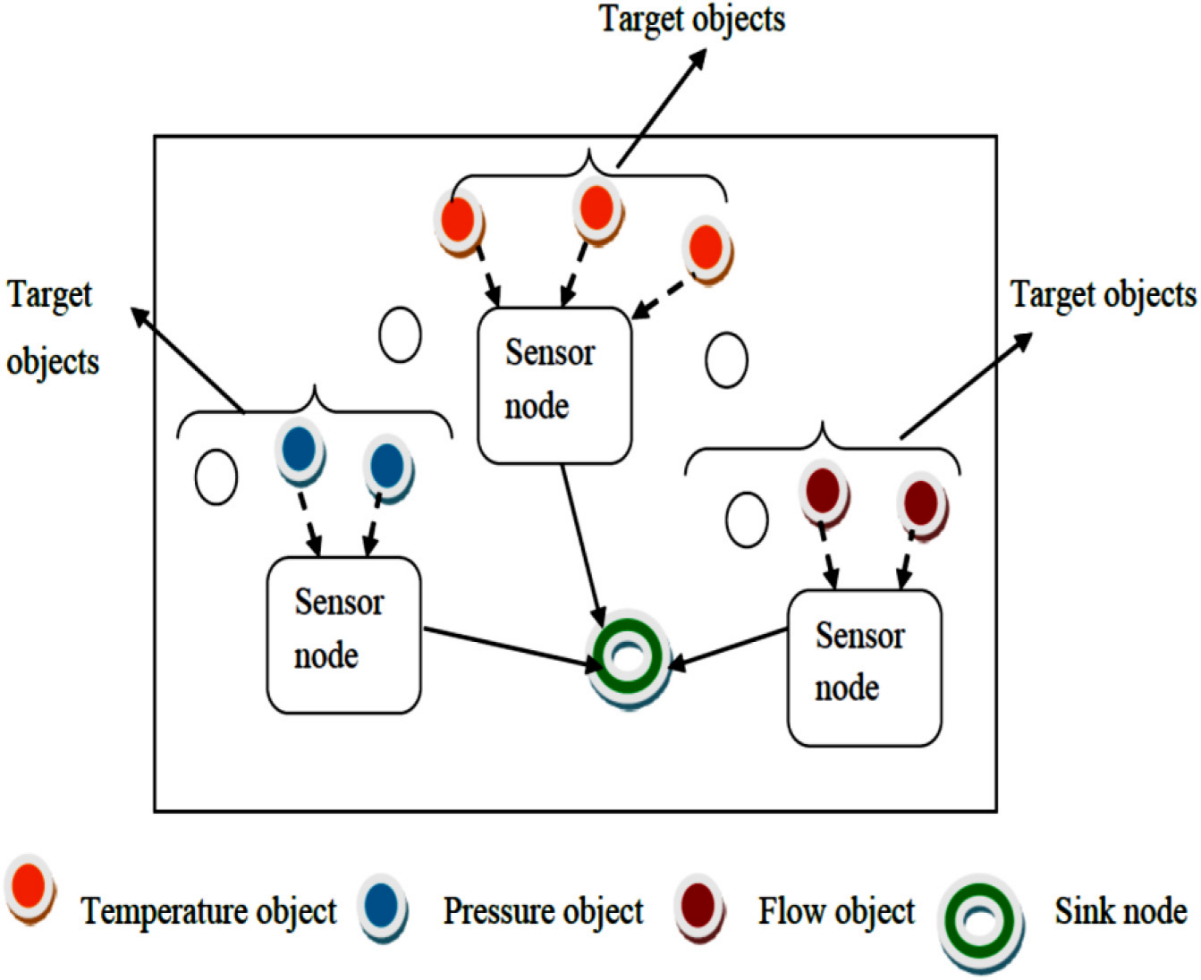
Object detection of similar event.

On the other hand, the BNEPD technique distributes the sensor nodes that detect an object of similar event (i.e., temperature, pressure, flow) into specific regions using Bayes principle which identifies minimal energy for sensed data being routed. Fig 2 shows the object detection of similar event using Bayes principle. As shown in Fig 2, the sensor nodes senses the data of corresponding frequency of temperature object, pressure object and flow object separately.

Fig 2 shows the object detection operation of similar event in WSN environment, where the sink node receives the task based on similar events (i.e., temperature, pressure etc). A node is identified as a target node that is generated through detection of object. The sink node receives numerous response packets from each source node, and then Bayes principle is applied on the sensor node organization with the objective of reducing energy consumption.

Let us consider the problem of routing using Bayes Node Energy Distribution and Polynomial Regression Function in Wireless Sensor Network in terms of a graph. If *G* = (*V*,*E*), then *V* denotes the set of all sensor nodes and *E* denotes the set of all communication stations connecting all sensor nodes in WSN. With this principle, the problem of routing is defined as the process of detection of object of similar event initiated at the source node and finally ending at all the destination nodes, based on the network frequency. This routing path detection of similar event is in the form a spanning tree *Tree* = (*V*,*E*) which includes the source sensor nodes and all target object nodes for which it is specifically made.

Bayes principle using BNEPD technique makes probabilities for each sensor node with the aid of probability models of corresponding frequency for which it is specifically made (i.e., temperature, pressure). In order to accomplish this, Bayes’ principle is used as a hypothesis for adjusting the degrees of belief (i.e., the presence of possibilities of temperature, pressure) given the observed evidence (i.e., target objects).

The Bayes principle in BNEPT technique obtains the probabilities of object detection of similar events. Then, the conditional probability concerning similar events is evaluated. The evaluated similar events are then sent to the sink node for further processing. The proposed Bayes principle for node energy distribution is then given as follows

$$Prob({TO}_{2}|{SN}_{1})=(\frac{\mathrm{Prob}({\mathrm{SN}}_{1}|{\mathrm{TO}}_{2})\;\mathrm{Prob}({\mathrm{TO}}_{2})}{\mathrm{Prob}({\mathrm{SN}}_{1})}$$ (1)

From (1), *SN*_1_ and *TO*_2_ are the sensor node and target object nodes respectively. Let *Dist*_*i*_ denotes the distance between the source and the target object nodes in WSN. Then the distance between *SN*_1_ and *TO*_2_ are measured as given below:

$${Dist}_{i}=\sqrt{\left({a_{1}-a_{2})}^{2}+(({b_{1}-b_{2})}^{2}\right)}$$ (2)

Based on the distance measurement given from (2), sensor nodes sense similar events of corresponding frequency (i.e., temperature, pressure and so on). In a similar manner, object detection of similar events is made based on the bayes probabilities and is sent to the sink node reducing the energy consumption.

### Polynomial Regression Function (Reduces Node Energy Drain Rate)

Upon successful completion of identification of objects of similar events by sensor nodes and sending it to the sink node, the task of sink node for data aggregation comes into action. The aggregation of data in BNEPD technique involves the process of collection and aggregation of similar events. With the objective of reducing the energy drain rate, data aggregation using polynomial regression function is used in the proposed work to save the limited resources through which the network lifetime is also increased and maintain high energy nodes in the routing path.

Data Aggregation using Polynomial Regression Function (DAPRF) in BNEPD technique represents the sensed objects in the form of polynomial functions. The idea behind BNEPD technique is that DAPRF performs aggregation of data with the aid of polynomial coefficients that represent sensor object.

Many sensor nodes send their objects (detected objects like pressure, temperature and so on) to the sink node. The sink node, on receiving the objects from multiple sensor nodes, then uses the Polynomial Regression Function to obtain the coefficient values of regression for each object. As a result, separate coefficient values are obtained for pressure, temperature and so on. The Polynomial Regression Function applied in BNEPD technique is given as below

$$PC=Func(a,b)=x_{0}+x_{1}b+x_{2}a+x_{3}ab+x_{4}abc$$ (3)

The ranges measured for each object isgiven by $\left(\begin{array}{c} a_{min},a_{max}(i.e.,object\;1),b_{min},b_{max}\\ \left(i.e.,object\;2\right) \end{array}\right)$.

Thus the minimum and maximum value of *a*, and *b* objects of similar events are calculated. The five regression values are then sent to the sink node, as well as the frequency of the objects of similar events for which the polynomial coefficients are evaluated. The sink node now has the following two sets of data (i.e., temperature and pressure) on receiving the coefficients from its target object of similar events:

$${Sink\;Node}_{i}=PC+{Range}_{FO}$$ (4)

$${Sink\;Node}_{i+1}=PC+{Range}_{SO}$$ (5)

From (4) and (5), the sink node ‘*i*’ th and ‘*i* + 1’ th value include the sum of polynomial coefficient of first object, coordinate range of first object of similar event, sum of polynomial coefficient of second object and finally the coordinate range of second object of similar event respectively.

The advantage of BNEPD technique is that the target object sends the polynomial coefficient obtained through polynomial regression function instead of sending the entire objects of similar events to the higher level. When a new target object is detected, an updated regression polynomial function is evaluated at higher level using polynomial coefficients sent by the objects of similar events and values obtained from the source object. As the BNEPD technique only sends the polynomial coefficient value, energy savings take place and therefore energy drain rate is reduced improving the performance of the entire network.

### Novel Distribution algorithm (Reduce Communication Overhead)

Finally, a novel distribution algorithm called Poly Distribute is designed to reduce the communication overhead. The Poly Distribute algorithm is distributed in such a way that it helps in the data aggregation improvement in BNEPD by designing the network in such a way that certain sensor nodes stop sensing and transmitting the data to the sink node for a time period. Finally, using Poly Distribute algorithm in BNEPD technique, the sink node uses Bayes principle on the object which it has received for the entire network. The algorithmic description of Poly Distribute applied in BNEPD technique is given below

#### Algorithm 1

Construction of Poly Distribute algorithm

Step 1: Determine the number of nodes to sense the target object of similar event

Step 2: Determine the number of nodes to be in sleep state

Step 3: Begin

Step 4: Repeat

Step 5: Detection of target objects by sensor nodes of similar event

Step 5.1: Apply Bayes principle

Step 5.2: Send objects of similar events to sink

Step 6: Perform data aggregation

 Step 6.1: Apply polynomial regression function

 Step 6.2: Send minimumand maximum value objects of similar events

 Step 6.3: Evaluate polynomial coefficient

Step 7: Until (all objects are attended)

Step 8: End

The above algorithm I shows the construction of poly distribute algorithm which performs three important steps. The first step involved in poly distribute algorithm is the selection of nodes. In this step, the number of nodes to be in the sleep state and the senor nodes to sense the target object of similar event is determined in a random manner. The random selection is designed on the basis of the number of nodes in the network and based on the remaining energy of each sensor nodes.

The second step is to detect the target object of similar events by the sensor nodes through Bayes principle based on the probability of events and distance function to the sink node. Finally, the sink node performs the data aggregation using polynomial regression function that determines the minimum and maximum value objects of similar events through polynomial coefficient. The above said process is repeated for all objects in the network. In this way, the communication overhead is reduced.

## Experimental Evaluation

In this section, we evaluate the proposed BNEPD technique and compare its performance to two other known data aggregation: Data Routing for In Network Aggregation (DRINA) for WSNs [1] and Cell Based Path Scheduling (CBPS) [2]. Simulations were conducted in NS2 (S1 File) using 350 node networks for various network scenarios randomly distributed in a 1000 square unit area.

Simulations were carried out in an extensive manner with random number for 70 iterations. To illustrate the simulation results for BNEPD technique, 350 sensor nodes were used with energy of each sensor node being 5 J, network size of 1000 * 1000 m with a transmission range of 100 m. We evaluate the BNEPD technique performance under the following metrics: Energy consumption, Energy drain rate, Communication overhead and time complexity.

Energy consumption using BNEPD technique is the product of sensor nodes, power (in terms of watts) and time (in terms of seconds). The energy consumption is measured in terms of Joules (J). The mathematical formulation for energy consumption using BNEPD technique is given as below

EC=No.sensornodes*Power*Time (6)

The energy drain rate for BNEPD technique is measured using exponential weighted moving average that represents the previous and newly calculated values. It is measured in terms of joules (J). The mathematical formulation for energy drain rate is given as below

$$EDR=No.sensor\;nodes\;*\;{EDR}_{old}\;*\;{EDR}_{new}$$ (7)

The communication overhead generated using BNEPD technique is the ratio of difference between actual communication time (i.e., between source node and target object of similar events) and computed communication time to actual communication time. It is measured in terms of milliseconds (ms). The mathematical formulation for communication overhead is given as below

$$CO=\frac{\left({Communication\;time}_{A}-{Communication\;time}_{C}\right)}{{Communication\;time}_{A}}$$ (8)

Time complexity using BNEPD technique is the time taken to sense the data or object of corresponding frequency. It is measured in terms of milliseconds (ms).

TC=Time(CO) (9)

The detailed simulation results of BNEPD technique with the aid of table values and graph is provided in the forthcoming sections.

## Simulation Results

In this section, simulation results are presented to evaluate the energy aware routing in wireless sensor network and to demonstrate the performance of the proposed distribution algorithm. In order to analyze the characteristics and functionality of the BNEPD technique, we quantitatively accessed the performance with the network size of 1000 * 1000 measured at 100 to 800 (m/s) using Dynamic Source Routing (DSR) Protocol by comparing the outcomes, the results achieved with the Poly Distribute algorithm. The Bayes Node Energy Polynomial Distribution (BNEPD) technique is compared against the existing Data Routing for In Network Aggregation (DRINA) for WSNs [1] and Cell Based Path Scheduling (CBPS) [2]. The experimental results using NS2 simulator are compared and analyzed using table values and graphical representations as given below.

### Impact of Energy Consumption

To support transient performance, in Table 1 we apply an efficient Poly Distribute algorithm to obtain the energy consumption rate and comparison is made with two other existing techniques, DRINA and CBPS.

**Table 1:** Tabulation for energy consumption.

No. of sensor nodes	Energy consumption (J)		
In numbers	BNEPD	DRINA	CBPS
50	0.51	0.58	0.63
100	1.23	1.35	1.44
150	1.85	2.05	2.23
200	2.52	2.82	3.15
250	3.49	3.78	4.25
300	3.42	3.55	4.01
350	4.28	4.87	5.13

The mathematical evaluation for energy consumption using BNEPD, DRINA and CBPS is given below. It is the product of number of sensor nodes, power consumed and time taken using three methods.

EC(usingBNEPD)=(50)*(1040*10−2*10−3)=0.52J (10)

EC(usingDRINA)=(50)*(1100*10−2*10−3)=0.55J (11)

EC(usingCBPS)=(50)*(1250*10−2*10−3)=0.625J (12)

**Fig 3:**
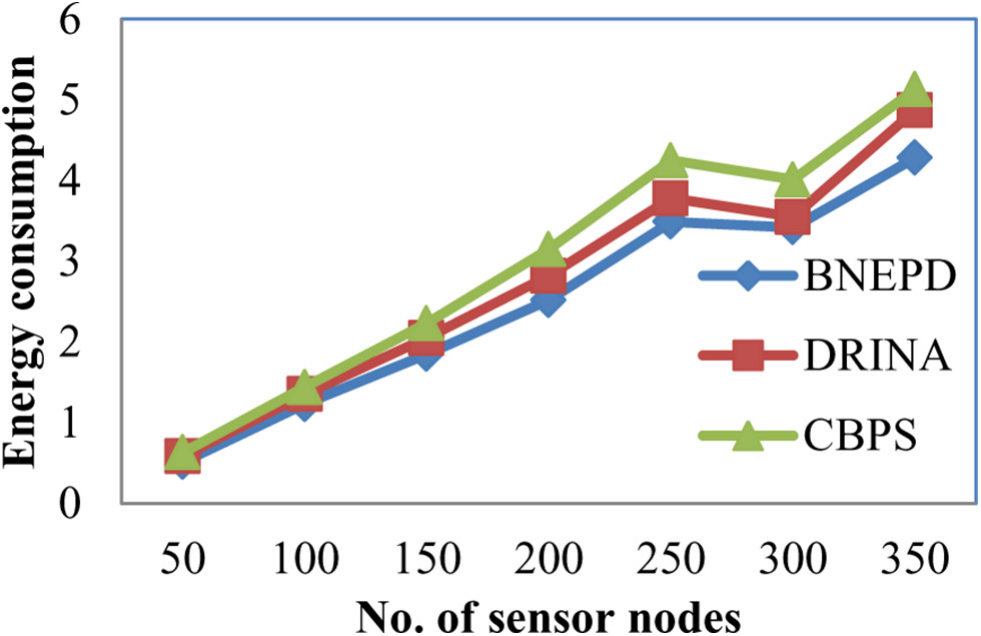
Measure of energy consumption.

Fig 3 depicts the energy consumption based on different sensor nodes. Our proposed BNEPD technique performs extensively well when compared to two other methods DRINA [1] and CBPS [2]. As illustrated in the Fig, the energy consumption is reduced than the two other methods with the application of Bayes Node Energy Distribution and had better changes when compared to the periodic aggregation applied in the existing technique and therefore provided reliable routing in Wireless Sensor Networks. In case of BNEPD technique, the Bayes Node Energy Distribution distributes the sensor nodes that detect an object of similar event into specific regions ensuring minimized energy consumption. By applying Bayes principle between source node and the target objects, for effective similar object detection, the energy consumption is reduced by 3–13% compared to DRINA and 17–23% compared to CBPS respectively.

### Measure of Energy Draining Rate

The Energy drain rate of our BNEPD technique is presented in Table 2. It is easy to find that the Energy drain rate is significantly reduced using BNEPD technique than the state-of-art methods.

**Table 2:** Tabulation for energy drain rate.

No. of sensor nodes	Energy drain rate (J)		
In numbers	BNEPD	DRINA	CBPS
50	8	10.5	13
100	9.5	11.2	14.5
150	10	13.4	15.8
200	11	14.2	17.3
250	12.5	15.8	19.5
300	14	17	20.2
350	15.5	17.8	22.4

The mathematical evaluation for energy drain rate using BNEPD, DRINA and CBPS is given below. It is the product of number of sensor node, old energy and new energy drain rate respectively.

EDR(usingBNEPD)=(50*0.5*0.3)=7.5J (13)

EDR(usingDRINA)=(50*0.5*0.4)=10J (14)

EDR(usingCBPS)=(50*0.5*0.5)=12.5J (15)

**Fig 4:**
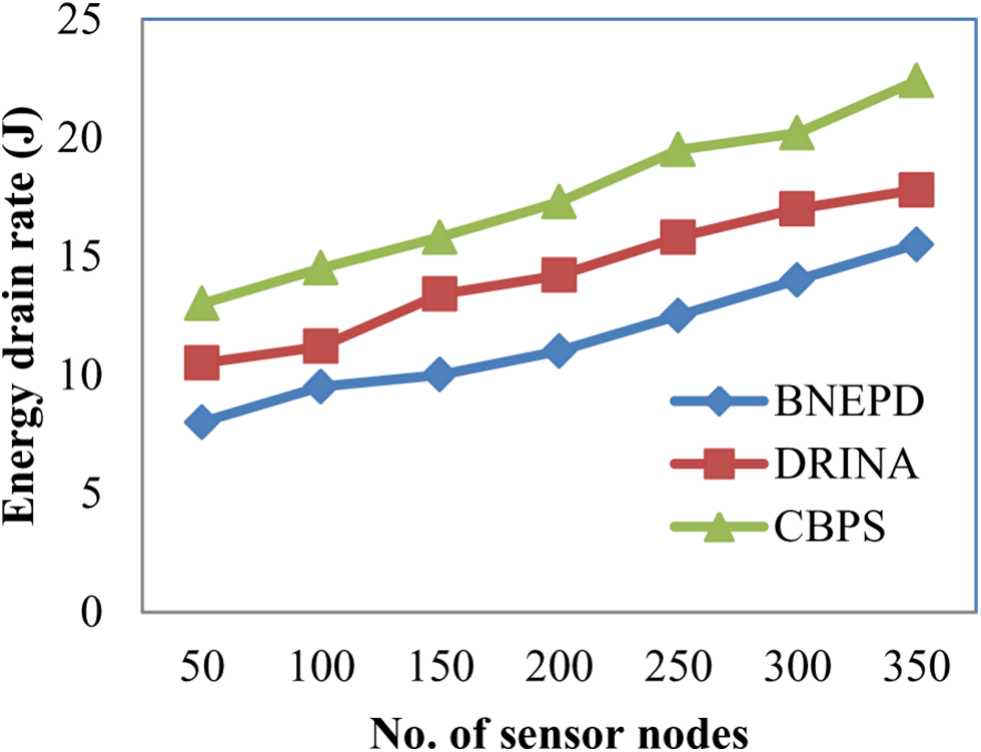
Measure of energy drain rate

Fig 4 shows the energy draining rate variations of 350 different nodes over a period of simulation time. Simulations are conducted through static and dynamic node positioning ranging from 50 to 350 nodes. From the Fig it is clear that as the number of sensor nodes increases the draining rate also increases. This is because of the application of polynomial regression function in BNEPD technique that maintains high energy nodes in the routing path with the objective of reducing the energy drain rate by 14–34% compared to DRINA and therefore improving the overall network. Also by applying the polynomial function, the coefficient values of the regression is obtained for each object. At the same time, separate coefficient values are obtained for pressure, temperature and so on, therefore reducing the energy drain rate by 44–62% compared to CBPS respectively.

### Impact of Communication Overhead

Table 3 describes the communication overhead over 350 sensor nodes. As the number of sensor nodes increases, the communication overhead is also increased. Comparison is also made with the existing FAF [12].

**Table 3:** Tabulation for communication overhead.

No. of sensor nodes	Communication overhead (ms)			
In numbers	BNEPD	DRINA	CBPS	FAF
50	0.27	0.41	0.45	0.52
100	0.35	0.44	0.55	0.60
150	0.41	0.48	0.59	0.63
200	0.38	0.43	0.55	0.64
250	0.40	0.44	0.58	0.66
300	0.43	0.47	0.61	0.68
350	0.41	0.44	0.56	0.65

Mathematical evaluation for communication overhead using BNEPD, DRINA and CBPS is given below. It is the difference between the actual communication time (i.e., ms) and computed communication time (i.e., ms). The communication overhead obtained using four methods are as given below.

CO(usingBNEPD)=(20–15)/20=0.25 (16)

CO(usingDRINA)=(20–12)/20=0.40 (17)

CO(usingCBPS)=(20–10)/20=0.50 (18)

CO(using FAF)=(20−8)/20=0.60 (19)

**Fig 5:**
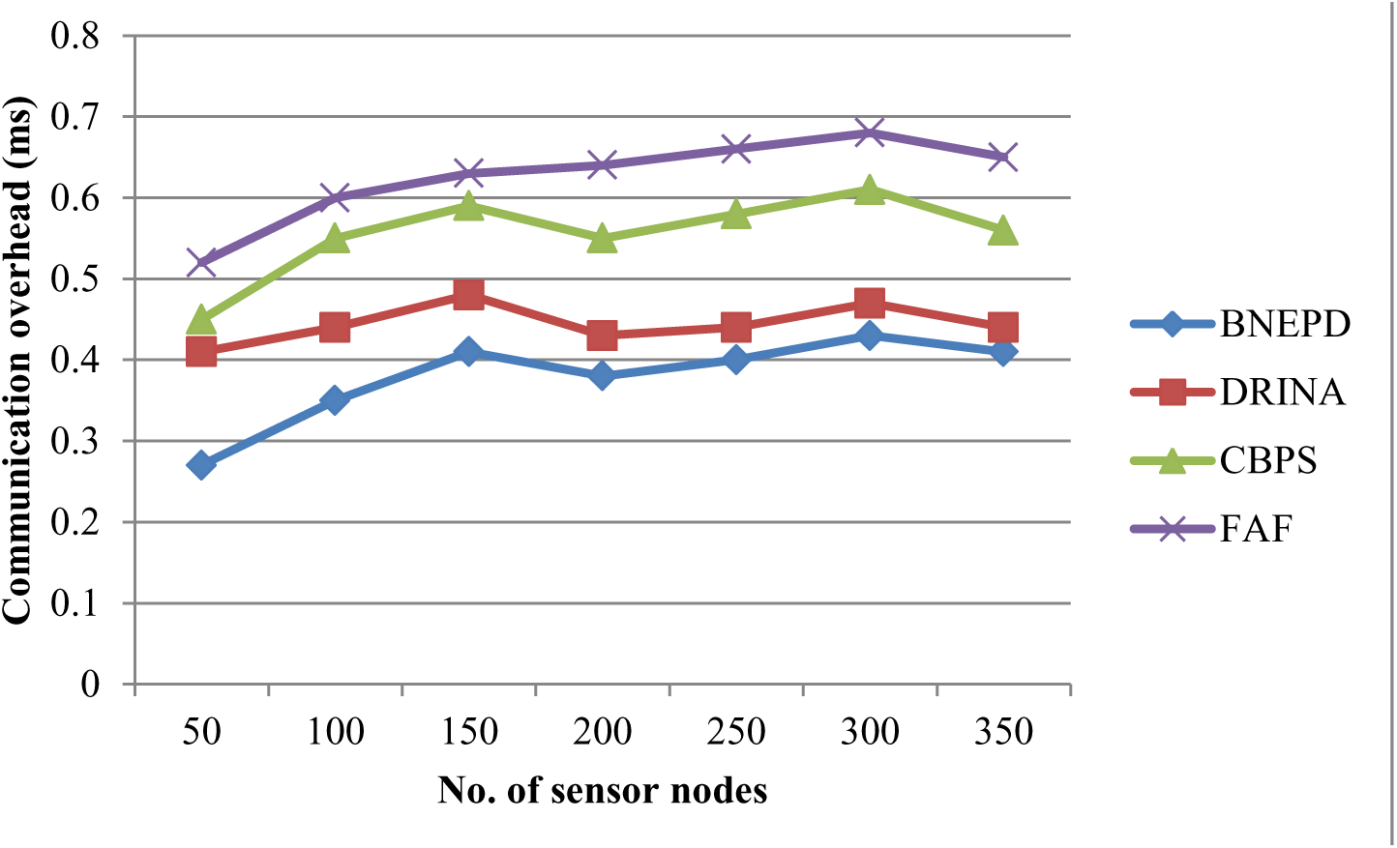
Measure of communication overhead

The targeting results of communication overhead using BNEPD technique with two state-of-the-art methods [1,2],[12] in Fig 5 is presented for visual comparison based on numerous sensor nodes. Our technique differs from DRINA [1] and CBPS [2] in that we have incorporated novel Poly Distribute Distribution algorithm that significantly reduce the communication overhead by stopping sensing and transmitting the data to the sink node for a time period. It then performs the mapping function which in turn reduces the communication overhead by 7–51% compared to DRINA. For the most different number of sensor nodes, the BNEPD technique, achieves comparable performance to Data Routing for In Network Aggregation (DRINA) for WSNs [1] and Cell Based Path Scheduling (CBPS) [2]. The storage overhead efficiency is improved by 36–66% when compared with the CBPS [2]. The communication overhead is reduced 46–92% as compared to existing FAF[12]

### Impact of Time Complexity

In Table 4 we further compare the time complexity of the proposed technique. Simulations were conducted using different number of sensor nodes which is measured in terms of milliseconds (ms).

**Table 4:** Tabulation for time complexity.

No. of sensor nodes	Time complexity (ms)		
In numbers	BNEPD	DRINA	CBPS
50	2.6	2.85	3.05
100	5.3	5.8	6.1
150	7.25	7.95	8.05
200	9.35	9.75	10.25
250	11.25	11.83	12.16
300	15.45	16.15	17.35
350	18.1	19.25	20.12

Mathematical evaluation for time complexity using BNEPD, DRINA and CBPS is given below. It is the time taken to perform the communication overhead for different number of sensor nodes. The time complexity using three methods are as given below.

TC(usingBNEPD)=50*0.050=2.5 (20)

TC(usingDRINA)=50*0.055=2.75 (21)

TC(usingCBPS)=50*0.058=2.9 (22)

**Fig 6:**
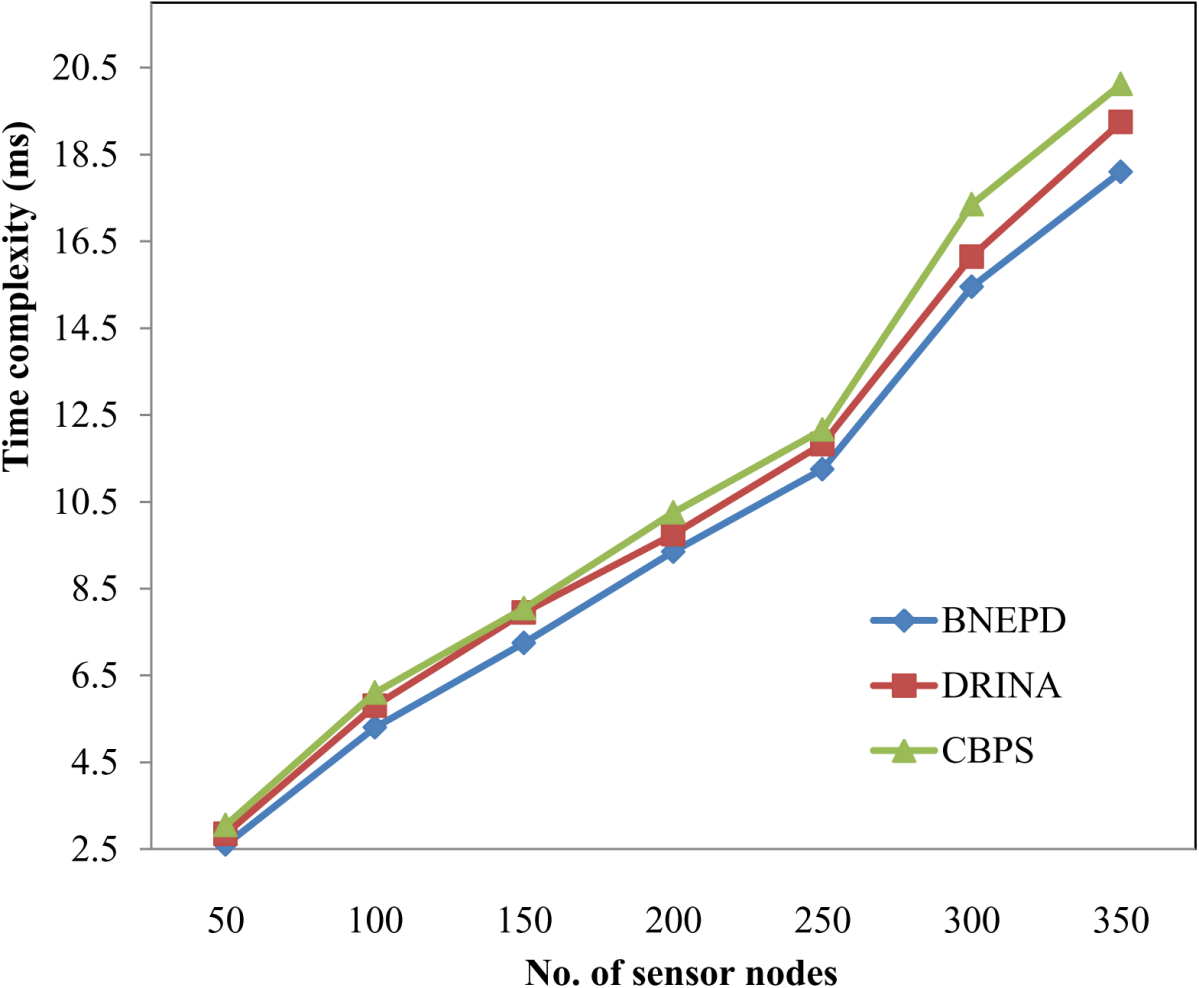
Measure of time complexity.

To explore the influence of time complexity on BNEPD technique, simulations are performed by applying 350 different sensors with a network size of 1000 * 1000 m in a transmission range of 100 ms depicted in Fig 6. The BNEPD technique shows competitive results compared to the state-of-the-art methods, namely DRINA [1] and CBPS [2]. The Fig also shows that the time complexity drastically reduces and reaches its zenith compared to two other methods because of the application of Bayes principle where sensor nodes efficiently detects target objects of similar events by sending it back to the sink nodes. In addition, efficient data aggregation at the sink nodes using polynomial regression function further improves the data aggregation efficiency with the help of polynomial coefficient using Poly Distribution algorithm. The application of Poly Distribution Algorithm efficiently helps in minimizing the time complexity by 4–9% when compared to DRINA [1] and 8–17% when compared to CBPS [2] respectively.

## Conclusion

We have proposed a new Bayes Node Energy Polynomial Distribution (BNEPD) technique to solve the issues related to routing and reduce energy drain rate with energy aware routing in Wireless Sensor Network. The energy aware routing in Wireless Sensor Network has been formulated as a Bayes Node Energy Distribution problem and solved through a novel probability method of object detection of similar events using novel Poly Distribute algorithms. The obtained results demonstrate better performance of objects of similar event detection and minimal energy for sensed data being routed over a number of sensor nodes. Our results also show that with the application of Bayes principle, node energy consumption is extensively reduced that effectively detects object of similar event and transmit the data to the sink node. Furthermore, the experimental part indicates that by applying polynomial regression function, the proposed BNEPD technique, converges minimized communication overhead based on the novel Poly Distribute algorithm that stops stop sensing and transmitting the data to the sink node for a time period. The performance of BNEPD technique is compared to other known data aggregation techniques. The performance of BNEPD technique was compared with different system parameters, and evaluated the performance in terms of different metrics, such as energy consumption, energy drain rate, time complexity and communication overhead. The results show that BNEPD technique offers comparatively better performance than the other techniques in all of the scenarios considered.

In the forthcoming research work, the proposed energy control for distributed estimation in wireless sensor networks needs to be expanding the wide range of network that uses more number of sensor nodes. This enables data to be stored and processed by devices with more resources.

## Supporting Information

S1 File The Network Simulator 2.33 (NS2) (An Open Source Simulator). (PDF)
