# Supplementary material for: Bayes Node Energy Polynomial Distribution to Improve Routing in Wireless Sensor Network
Source: PLoS One. 2015 Oct 1;10(10):e0138932. doi: 10.1371/journal.pone.0138932 (PMC4591332; doi:10.1371/journal.pone.0138932)
Supplement: S1 File — (PDF) [file pone.0138932.s001.pdf]

The Network Simulator 2.33 (NS2)

(An Open Source Simulator)

Network Simulator (NS2) is a discrete event driven simulator developed at UC Berkeley. It is part of the VINT project. The goal of NS2 is to support networking research and education. It is suitable for designing new protocols, comparing different protocols and traffic evaluations. NS2 is developed as a collaborative environment. It is distributed freely and open source. A large amount of institutes and people in development and research use, maintain and develop NS2. This increases the confidence in it. Versions are available for FreeBSD, Linux, Solaris, Windows and Mac OS X.

Functionalities of NS 2.33

Functionalities for wired, wireless networks, tracing, and visualization are available in NS2.

• Support for the wired world include

- Routing DV, LS, and PIM-SM.
- Transport protocols: TCP and UDP for unicast and SRM for multicast.
- Traffic sources: web, ftp, telnet, cbr (constant bit rate), stochastic, real audio.
- Different types of Queues: drop-tail, RED, FQ, SFQ, DRR.
- Quality of Service: Integrated Services and Differentiated Services.
- Emulation.

• Support for the wireless world include

- Ad hoc routing with different protocols, e.g. AODV, DSR, DSDV, TORA
- Wired-cum-wireless networks
- Mobile IP
- Directed diffusion

- Satellite
- Senso-MAC
- Multiple propagation models (Free space, two-ray ground, shadowing)
- Energy models

• Tracing

• Visualization

- Network Animator (NAM)
- Trace Graph

• Utilities

- Mobile Movement Generator

Mobile Networking in NS2.33

This section describes the wireless model that was originally ported as CMU's Monarch group's mobility extension to NS2. The first section covers the original mobility model ported from CMU/Monarch group. In this section, we cover the internals of a mobile node, routing mechanisms and network components that are used to construct the network stack for a mobile node. The components that are covered briefly are Channel, Network interface, Radio propagation model, MAC protocols, Interface Queue, Link layer and Address resolution protocol model (ARP).

CMU trace support and Generation of node movement and traffic scenario files are also covered in this section. The original CMU model allows simulation of pure wireless LANs or multihop ad-hoc networks. Further extensions were made to this model to allow combined simulation of wired and wireless networks. MobileIP was also extended to the wireless model.

The Basic Wireless Model in NS

The wireless model essentially consists of the MobileNode at the core, with additional supporting features that allows simulations of multi-hop ad-hoc networks, wireless LANs etc. The MobileNode object is a split object. The C++ class MobileNode is derived from parent class

Node. A MobileNode thus is the basic Node object with added functionalities of a wireless and mobile node like ability to move within a given topology, ability to receive and transmit signals to and from a wireless channel etc. A major difference between them, though, is that a MobileNode is not connected by means of Links to other nodes or mobilenodes. In this section we shall describe the internals of MobileNode, its routing mechanisms, the routing protocols dsdv, aodv, tora and dsr, creation of network stack allowing channel access in MobileNode, brief description of each stack component, trace support and movement/traffic scenario generation for wireless simulations.

Mobile Node: Creating Wireless Topology

Mobile Node is the basic ns Node object with added functionalities like movement, ability to transmit and receive on a channel that allows it to be used to create mobile, wireless simulation environments. The class Mobile Node is derived from the base class Node. Mobile Node is a split object. The mobility features including node movement, periodic position updates, maintaining topology boundary etc are implemented in C++ while plumbing of network components within Mobile Node itself (like classifiers, dmux , LL, Mac, Channel etc) have been implemented in Otcl.

ARCHITECTURE OF NS-2

NS is written in C++, with an OTcl1 interpreter as a command and configuration interface. The C++ part, which is fast to run but slower to change, is used for detailed protocol implementation. The OTcl part, on the other hand, which runs much slower but can be changed very fast quickly, is used for simulation configuration. One of the advantages of this split-language program approach is that it allows for fast generation of large scenarios. To simply use the simulator, it is sufficient to know OTcl. On the other hand, one disadvantage is that modifying and extending the simulator requires programming and debugging in both languages.

NS can simulate the following:

- Topology: Wired, wireless
- Sheduling Algorithms: RED, Drop Tail,

- ❖ Transport Protocols: TCP, UDP
- ❖ Routing: Static and dynamic routing
- ❖ Application: FTP, HTTP, Telnet, Traffic generators

USER'S VIEW OF NS-2

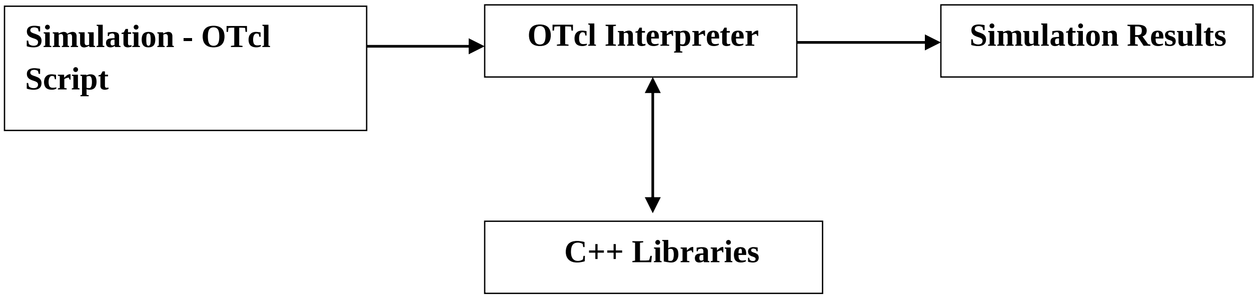

Fig 1: Block diagram of Architecture of NS-2
